# CpG methylation of *MGMT* and *hMLH*1 promoter in hepatocellular carcinoma associated with hepatitis viral infection

**DOI:** 10.1038/sj.bjc.6600743

**Published:** 2003-02-18

**Authors:** S Matsukura, H Soejima, T Nakagawachi, H Yakushiji, A Ogawa, M Fukuhara, K Miyazaki, Y Nakabeppu, M Sekiguchi, T Mukai

**Affiliations:** 1 Department of Biochemistry, Saga Medical School, 5-1-1 Nabeshima, Saga 849–8501, Japan; 2 Department of Surgery, Saga Medical School, 5-1-1 Nabeshima, Saga 849–8501, Japan; 3 Department of Pathology, Saga Medical School, 5-1-1 Nabeshima, Saga 849–8501, Japan; 4 Department of Biochemistry, Medical Institute of Bioregulation, Kyushu University, 3-1-1 Maedashi, Higashi-ku, Fukuoka 812–8582, Japan; 5 Division of Neurofunctional Genomics, Medical Institute of Bioregulation, Kyushu University, 3-1-1 Maedashi, Higashi-ku, Fukuoka 812–8582, Japan; 6 Core Research for Evolutional Science and Technology, (CREST) of Japan Science and Technology Corporation, Japan; 7 Department of Biology and Frontier Research Center, Fukuoka Dental College, 2-15-1 Tamura, Sawara-ku, Fukuoka 814–0193, Japan

**Keywords:** CpG methylation, MGMT, *hMLH1*, hepatocellular carcinoma, hepatitis viral infection, urea/bisulphite sequencing

## Abstract

Inactivations of DNA repair genes, *O*^6^-*methylguanine-DNA methyltransferase (MGMT)* and *hMLH1*, by promoter hypermethylation have been reported in several types of primary human neoplasia. This epigenetic inactivation mechanism remains elusive in hepatocellular carcinoma (HCC). To investigate the relation between the expression of *MGMT* and *hMLH1* and the CpG methylation within their promoters in HCCs with or without hepatitis viral infection, we performed immunohistochemistry and urea/bisulphite sequencing on 46 HCCs, corresponding noncancerous tissues, and 20 normal liver tissues. MGMT- and hMLH1-negative HCCs were 60.9% (28 out of 46) and 21.8% (10 out of 46), respectively. HCCs lacking both proteins were 10.9% (five out of 46). The frequency and extent of CpG methylation in the *MGMT* promoter increased along with hepatitis viral infection and pathological progression. MGMT-negative tumours showed very frequent and widespread methylation in the promoter compared with MGMT-positive tumours. Half of the hMLH1-negative HCCs showed promoter hypermethylation. These data suggested that *MGMT* gene silencing in a subset of HCCs was likely caused by epigenetic alteration, such as promoter hypermethylation, and that the promoter hypermethylation silenced the *hMLH1* gene in half of the hMLH1-negative tumours. A correlation between the promoter methylation status and viral infection, although it was weak, intimated that hepatitis viral infections could play a role in the CpG methylation of the *MGMT* promoter.

Hepatocellular carcinoma (HCC) is one of the most frequent human malignancies and a major cause of cancer-related deaths worldwide (Okuda *et al*, 1992). Most HCCs exhibit characteristics compatible with chronic hepatitis and cirrhosis caused by persistent infection of hepatitis B virus (HBV) and/or hepatitis C virus (HCV) (Okuda *et al*, 1992). Both chronic hepatitis and cirrhosis associated with viral infection are considered as precancerous conditions (Paradis *et al*, 1998). The process of chronic inflammation or cirrhosis initiates clonal expansion and facilitates regeneration of hepatocytes (Aihara *et al*, 1994). During neoplastic degenerative change, an accumulation of genetic alterations or epigenetic changes may occur. However, the molecular mechanisms of hepatocarcinogenesis associated with hepatitis viral infection have not been clarified.

*O*^6^-methylguanine-DNA methyltransferase (MGMT) is a DNA repair enzyme that plays an important role in the defence against the carcinogenic and cytotoxic effects of alkylating agents in cellular DNA (Pegg, 1990). Since ubiquitous and environmental alkylating agents such as *N*-nitroso compounds are principally metabolised and activated in mammalian hepatocytes, liver tissue is persistently exposed to activated alkylating agents (Gerson *et al*, 1986). It was demonstrated that *Mgmt*-targeted mice (*Mgmt*^−/−^) treated with alkylating agents generated hepatocellular carcinoma (Iwakuma *et al*, 1997). Major and Collier (1998) suggested that MGMT protein activity decreased in chronic hepatitis, cirrhosis, and HCCs. We have recently reported that the MGMT expression was frequently lost in a variety of human tumours and was a significant prognostic factor (Matsukura *et al*, 2001). Since loss of MGMT expression was not commonly because of a genetic change, it has been suggested that another cause, such as epigenetic change, is involved (Esteller *et al*, 1999; Bhakat and Mitra, 2000).

Mismatch repair system (MMR) is an essential system by which cells correct errors in DNA replication during proliferation to maintain the fidelity of the genome (Lahue *et al*, 1989). One of the MMR genes, *hMLH1*, has been demonstrated to play a pivotal role in DNA MMR (Papadopoulos *et al*, 1994). In addition, the important association between MGMT and MMR in DNA repair was pointed out by Frayling (1999). Furthermore, it was shown that *Mgmt*^−/−^ *Mlh1*^−/−^ (double knockout) mice treated with alkylating agents exhibited high susceptibility to carcinoma (Kawate *et al*, 1998). It is, however, unclear whether tumours expressing neither of the genes exist in human HCCs.

It has been proposed that aberrant DNA methylation of CpG islands in the promoter region is correlated with inactivation of tumour suppressor genes in human cancer. Esteller *et al*. (2001) have demonstrated the reduced expression of tumour suppressor genes such as *p16*, *MGMT*, and *hMLH1* by promoter hypermethylation in several human neoplasias, and have suggested that this epigenetic change might be an early event in the pathogenesis of several human tumours. Recently, the correlation of *p16* promoter hypermethylation with chronic hepatitis and cirrhosis associated with HBV or HCV infection has been reported (Kaneto *et al*, 2001).

Here, we report the existence of human HCCs lacking both MGMT and hMLH1 proteins, the relation between HCCs associated with hepatitis viral infection and detailed CpG methylation status of *MGMT* and *hMLH1* promoter regions, and the specific CpG methylation pattern of MGMT- and hMLH1-negative tumours.

## MATERIALS AND METHODS

### Tissue specimens

A total of 46, HCCs and adjacent noncancerous liver tissues (mean age 63.8 years; 35 males and 11 females; seven HBV positives, 33 HCV positives, one HBV/HCV positive and five HBV/HCV negatives) and 20 normal liver (mean age 59.9 years; 14 males and six females) tissue specimens were obtained surgically and frozen at −80°C. All specimens were subjected to pathological diagnosis. DNAs of all tumour samples were extracted from pathologically obvious cancerous regions in the resected liver. HBV and HCV infections were diagnosed serologically with HBs antigen (LPIA-200; Diatron Laboratories, Tokyo, Japan) and anti-HCV antibody (Immunocheck-HCV Ab; International Reagent, Kobe, Japan), respectively. Informed consent was obtained from all patients.

### Anti-MGMT antibody

Polyclonal rabbit antibodies against human MGMT protein were prepared using TrpE fusion protein, as described (Nakabeppu and Nathans, 1991). *Escherichia coli* BL21 (DE3) carrying pET3d:TrpE-hMGMT-1 that encodes the TrpE polypeptide fused to a region of MGMT (residues 1–45) at the C terminus was used to produce each fusion protein (Studier *et al*, 1990), and polyclonal antibodies against the fusion protein were raised in rabbits. The serum was initially applied to a TrpE-hMGMT-1-coupled column; their bound materials were eluted at pH 2.3 and dialysed against 10 mM Tris-HCl (pH 7.4) and 150 mM NaCl. To increase antibody specificity, the eluted fraction was applied to an affinity column with TrpE-mMGMT-1, in which a corresponding region of mouse MGMT (residues 1–58) was fused to TrpE (Kawate *et al*, 1995), as a ligand. Then the bound fraction was eluted and dialysed. This fraction was used as an anti-MGMT antibody. The specificity of the antibody has been reported previously (Matsukura *et al*, 2001).

### Immunohistochemistry

Immunohistochemical studies for MGMT (Matsukura *et al*, 2001) and hMLH1 (Wang *et al*, 2001) were performed as described previously. In the present study, mouse monoclonal antibody against hMLH1 protein (clone G168-728; PharMingen, San Diego, CA, USA) (Wang *et al*, 2001) was used. Positive staining was identified by the presence of brown staining in the nucleus and/or cytoplasm. MGMT and hMLH1 expression were evaluated as positive if the distribution of stained cells was more than 10% of cancer cells. The expression status of MGMT and hMLH1 was assessed by two pathologists without a knowledge of the clinicopathological features of the case or the clinical outcome.

### Urea/bisulphite modification of DNA and PCR amplification

**Figure 2:**
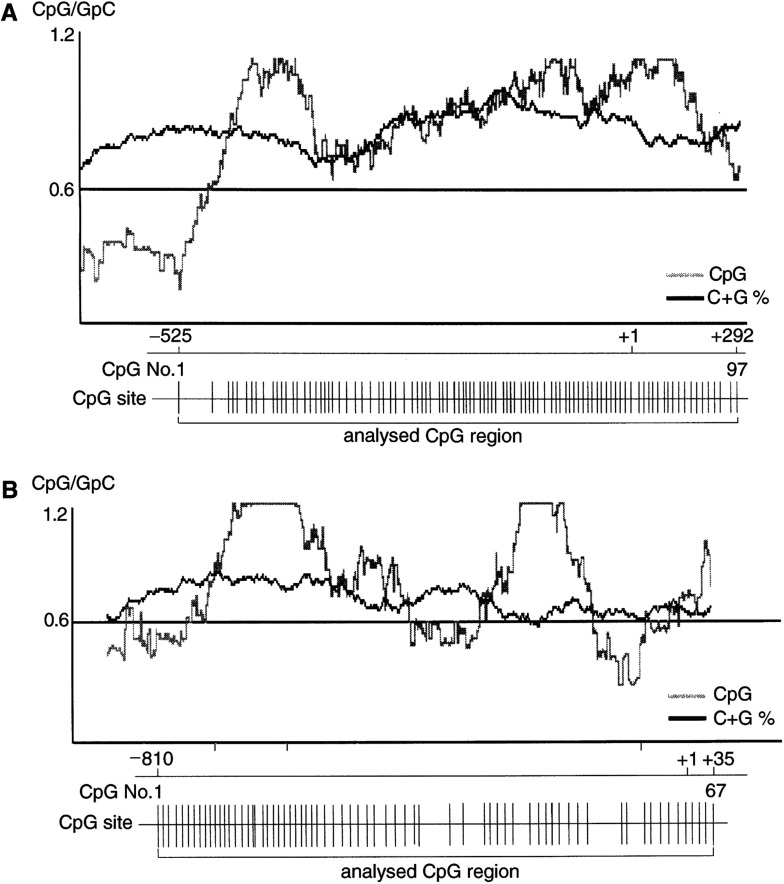
CpG island of the *MGMT* and *hMLH1* promoter region. (**A**) Schematic representation of the *MGMT* CpG island containing 97 CpG sites, spanning −525 nt to +292 nt, relative to the transcriptional start site (+1). (**B**) Schema of the CpG island in *hMLH1* containing 67 CpG sites, spanning −810 nt to +35 nt. Vertical bars denote the location of each CpG site. The graph shows the CpG island defined by Gardiner-Garden and Frommer of a region greater than 200 bp with a high GC content (grey line, number of CpG; black line, the frequency of C+G) and an observed/expected ratio for the occurrence of CpG more than 0.6.

The urea/bisulphite treatment of genomic DNA was performed as described by Paulin *et al* (1998). The modified DNA was resuspended in 20 *μ*l of water and immediately subjected to PCR or stored at –20°C. The whole CpG island in the *MGMT* promoter region was amplified by nested PCR (Figure 2A

). Primer sequences and PCR conditions are presented in Table 1

**Table 1:** Primer sequence and PCR conditions

	**Primer sequences**	**PCR cycles**
*MGMT*		
1st PCR		
S1	TTGGAIGGTATIGTTATTATAGG	40 cycles of 95°C/30 s, 50°C/30 s, 72°C/90 s
AS1	AATAAATAAAAATCAAAACIACCC	
2nd PCR		
S2	TTATTATAGGTTTTGGAGGTTGTTT	30 cycles of 95°C/30 s, 60°C/30 s, 72°C/60 s
AS2	AAAAATCAAAACIACCCCCC	
		
*hMLH1*		
1st PCR		
S1	GAIGTTTATATGTTIGGGTAGTAT	40 cycles of 95°C/30 s, 50°C/30 s, 72°C/90 s
AS1	ACCACIAACIACATTTTAACICC	
2nd PCR		
S2	TTTTTATTTTAGTIGIGATTTTTTA	30 cycles of 95°C/30 s, 52°C/30 s, 72°C/60 s
AS2	CAAAAAAACCAAAAAAACITCTAAA	
		
*Colony PCR*		
VS1	TCCGGCTCGTATGTTGTGTGGA	20 cycles of 96°C/20 s, 60°C/20 s, 72°C/120s
VAS1	GTGCTGCAAGGCGATTAAGTTGG	

I=inosine.

. The first round of amplification was performed with 50 ng of the bisulphite-treated DNA. Then 1/1000th of the first PCR product was subjected to the second round PCR. The size of the nested PCR product was 835 bp. The CpG island of *hMLH1* was also amplified by nested PCR (Figure 2B). The size of the nested PCR product was 815 bp.

### Cloning of PCR product and sequencing

The amplicons were cloned into the pSTBlue-1 Acceptor™ Vector (Novagen Inc., Madison, WI, USA) to transform competent JM109 cells (Wako Pure Chemical Industries, Ltd, Osaka, Japan). The plasmid DNAs were amplified by colony PCR reactions (Table 1). A total of 10 of the colony PCR products from each sample were sequenced.

### Statistical analysis

Differences among groups (normal liver tissues *vs* chronic hepatitis, liver cirrhosis and HCC, or MGMT-negative *vs* -positive HCCs) were tested by one-way analysis of variance (ANOVA). Probability levels of <0.05 were considered statistically significant.

## RESULTS

### Immunohistochemistry of MGMT and hMLH1

**Figure 1:**
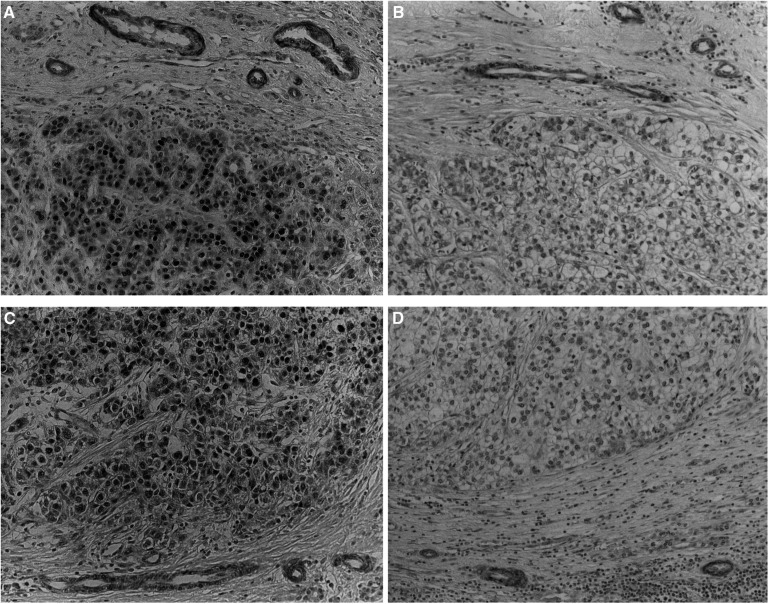
Immunohistochemical staining of MGMT (**A**, **B**) and hMLH1 (**C**, **D**) in HCCs. MGMT- or hMLH1-positive tumour cells showed strongly stained nuclei (**A**, **C**), while no stained nucleus was detected in MGMT- or hMLH1-negative tumours (**B**, **D**), except for bile duct cells as an internal positive control.

Immunohistochemical analysis was performed on normal liver, noncancerous tissues, and HCCs to examine whether MGMT and hMLH1 were expressed. Expression of these proteins was detected in cells from normal liver, as well as in chronic hepatitis and liver cirrhosis, including hepatocytes, bile duct cells, vascular endothelial cells, smooth muscle, and so on (data not shown). We used bile duct cells as an internal positive control within the sections because these cells in cancerous regions also commonly expressed MGMT and hMLH1 (Figure 1B, D

). The signals of MGMT-positive tumour cells were as intense as those of the bile duct cells (Figure 1A), while those of MGMT-negative tumour cells were virtually undetectable (Figure 1B). As for hMLH1, signal intensity similar to those of bile duct cells was detected in hMLH1-positive tumour cells (Figure 1C), but not in hMLH1-negative tumours (Figure 1D). MGMT- and hMLH1-negative tumours were 60.9% (28 out of 46) and 21.8% (10 out of 46), respectively. Tumours lacking both proteins were 10.9% (5 out of 46) of all tumours. The results of immunohistochemistry and the characteristics of patients with HCC are shown in Table 2

**Table 2:** Background of patients with hepatocellular carcinomas

**Clinical parameters**				**Pathological parameters of tumours**			**Immunohistochemistry**	
**Case**	**Age**	**Sex**	**Viral status**	**Noncancerous liver tissue**	**Differentiation (grade)**	**Size (cm)**	**MGMT**	**hMLH1**
1	62	M	C	CH	I	5.5	–	+
2	58	M	B	CH	II	10.3	–	+
3	73	F	C	CH	II	6.3	–	+
4	60	M	B & C	LC	I	2.0	–	+
5	62	F	C	LC	III	2.8	–	+
6	48	M	B	LC	II	6.0	–	+
7	51	M	B	LC	I	4.5	–	+
8	58	F	C	CH	II	8.5	–	–
9	67	M	C	CH	III	2.1	–	+
10	51	M	NBNC	N	II	2.5	–	–
11	68	M	C	LC	II	2.0	–	+
12	66	M	C	CH	II	4.5	–	–
13	72	M	C	LC	II	10.5	–	+
14	57	M	B	LC	III	3.0	–	+
15	67	M	C	CH	II	2.0	–	+
16	62	M	NBNC	N	I	6.0	–	+
17	65	M	C	LC	II	2.4	–	–
18	51	M	C	CH	II	4.5	–	+
19	54	M	B	LC	III	4.3	–	+
20	65	M	C	CH	II	8.5	–	+
21	73	M	C	LC	II	3.0	–	+
22	61	M	C	CH	II	3.0	–	+
23	59	M	C	LC	I	3.5	–	–
24	71	M	C	LC	I	9.0	–	+
25	68	M	C	CH	II	2.5	–	+
26	64	M	C	CH	III	2.0	–	+
27	74	M	C	CH	I	7.0	–	+
28	53	M	NBNC	N	I	6.0	–	+
29	72	F	C	LC	II	2.8	+	–
30	70	M	NBNC	N	II	14.0	+	+
31	63	M	C	CH	II	3.2	+	+
32	50	M	B	LC	II	4.0	+	+
33	60	F	C	LC	I	2.5	+	+
34	60	M	NBNC	N	I	3.0	+	–
35	70	M	C	CH	II	4.0	+	–
36	65	F	C	CH	II	2.5	+	–
37	70	F	C	CH	II	4.2	+	–
38	68	M	C	LC	II	3.5	+	+
39	73	F	B	CH	II	3.0	+	+
40	66	M	C	CH	I	4.0	+	+
41	62	M	C	LC	II	3.6	+	+
42	62	M	C	LC	II	2.5	+	+
43	69	F	C	CH	II	8.5	+	+
44	68	F	C	LC	II	3.5	+	+
45	70	F	C	CH	II	2.0	+	+
46	71	M	C	CH	I	2.0	+	+

Viral status B=HBs Ag positive; C=HCV antibody positive; NBNC=HBs Ag and HCV antibody negative; CH=chronic hepatitis; LC=liver cirrhosis; N=normal liver; grade I=well-differentiated HCC; grade II=moderately differentiated HCC; grade III=poorly differentiated HCC; +=positive; −=negative.

.

### Methylation analysis of *MGMT* promoter region in HCCs, noncancerous, and normal liver tissues

Next, we performed urea/bisulphite sequencing to investigate the methylation status of *MGMT* and *hMLH1* promoter region in HCCs, noncancerous tissues, and normal liver. Prior to the study of primary samples, to confirm that the nested PCR we used could accurately reflect the methylation status of genomic DNA, we performed PCR using DNA solutions containing methylated and unmethylated genomic DNAs with different ratios. The methylated and unmethylated DNAs were extracted from an MGMT-deficient cell line, SW48 (Aquilina *et al*, 1998), in which the MGMT promoter was fully methylated and an MGMT-proficient cell line, HepG2 (Fritz and Kaina, 1992), in which the promoter was not methylated at all. We found that the ratio of methylated to unmethylated clones obtained from the nested PCR–cloning–sequencing was consistent with the theoretical ratio (data not shown).

**Figure 3:**
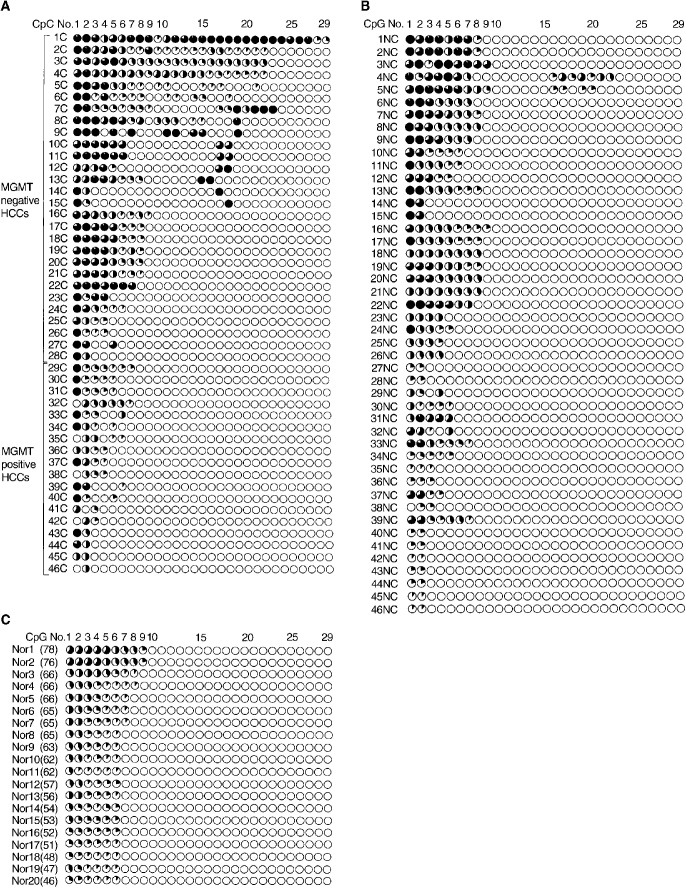
Detailed CpG (No. 1–29) methylation status of all analysed 97 CpGs within the *MGMT* promoter region in HCCs (**A**), adjacent tissues (**B**), and normal liver (**C**). Each circle graph represents the percentage of methylated clones (number of methylated clones/10 analysed clones×100). Age in parentheses (**C**).

The CpG island of *MGMT* including 97 CpG sites is shown in Figure 2A. We examined the detailed methylation status of all CpG sites by urea/bisulphite DNA sequencing in 46 HCCs (Figure 3A

), corresponding noncancerous tissues (Figure 3B), and 20 normal liver tissues (Figure 3C). The methylation was somewhat observed at the 5′ border of the CpG island irrespective of cancerous or noncancerous samples. These methylated CpGs in MGMT-positive tumours, noncancerous tissues except for 4NC and 5NC, and normal tissues never extended beyond the first nine CpGs. However, a subset of MGMT-negative HCCs, 1C–15C, showed that the methylation extended in the 3′ direction beyond the first nine CpGs. Tumour 1C showed all 97 hypermethylated CpG sites in the island (data not shown). Other MGMT-negative tumours, 16C–28C, showed a similar extent of methylation to MGMT-positive tumours and normal tissues.

**Figure 4:**
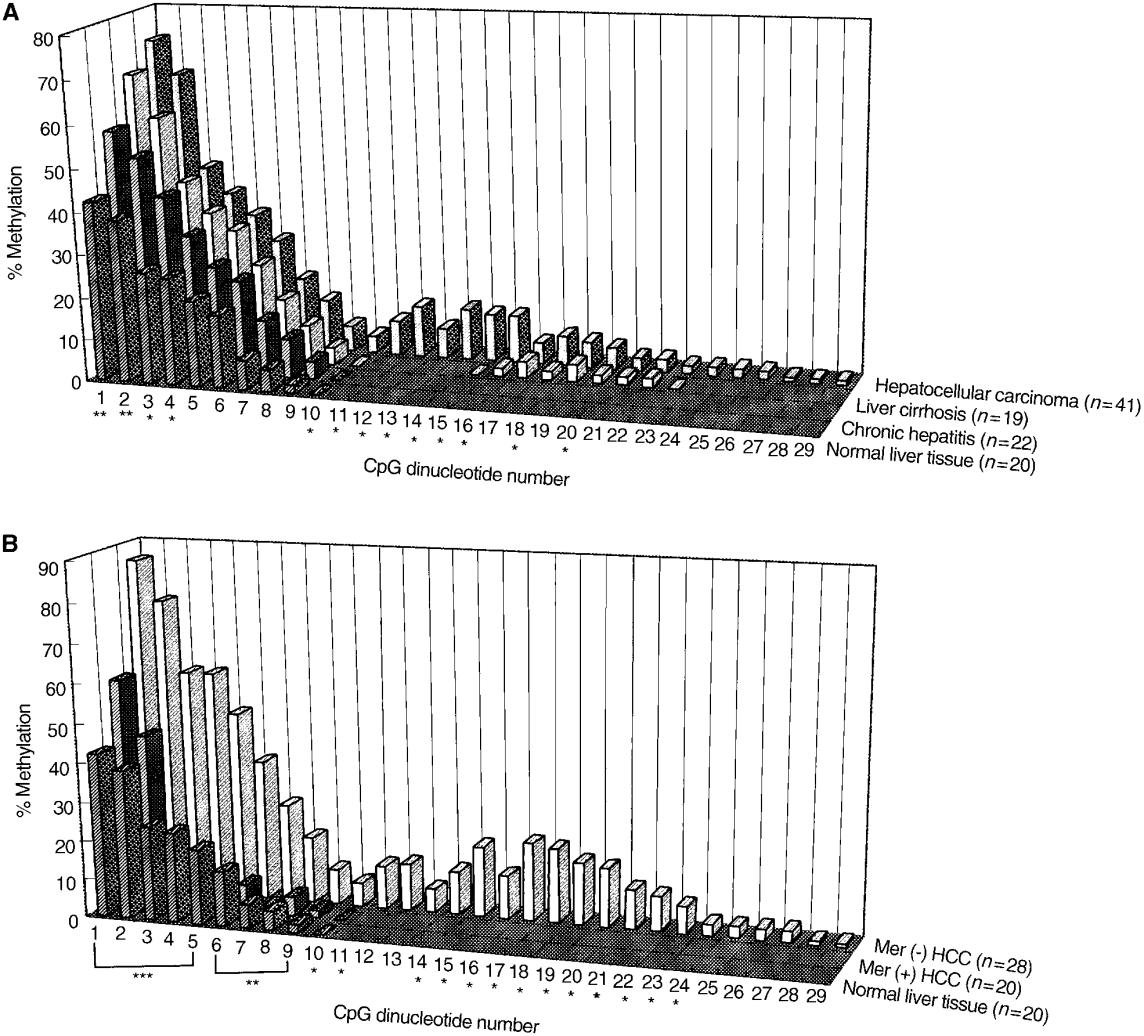
Frequency of methylation (%) of CpG sites (No. 1–29) in MGMT promoter region. (**A**) Methylation frequency of HCCs, liver cirrhosis, chronic hepatitis with hepatitis viral infection, and normal liver tissues without infection were compared. ^*^P<0.05 and ^**^P<0.01 indicate the significant differences of methylation frequency of each pathological stage *vs* that of normal liver at the CpG site. (**B**) Methylation frequencies of MGMT-negative (Mer(−)) HCCs, MGMT-positive (Mer(+)) HCCs, and normal liver tissues were compared. Frequency was calculated by (total number of methylated clones/10 analysed clones×case number at each CpG site)×100. ^*^P<0.05, ^**^P<0.01, and ^***^P<0.001 indicate the significant differences of methylation frequency of Mer (+) HCC *vs* Mer (−) HCC at CpG site.

From the point of view of hepatitis viral infection, the methylation frequency at particular CpG sites in chronic hepatitis, liver cirrhosis, and HCC was higher than that in normal liver with statistical significance (Figure 4A

). The frequency at those sites increased along with pathological progression. Furthermore, the methylation also extended towards downstream along with pathological progression (Figure 4A). These data suggested that hepatitis viral infection might be involved in methylation of the MGMT promoter region. As for MGMT expression, MGMT-negative tumours showed high frequency and widespread methylation compared with MGMT-positive tumours and normal liver (Figure 4B). There were significant statistical differences in the methylation frequency at many CpG sites between MGMT-negative and -positive HCCs. This result suggested that hypermethylation of the MGMT promoter might play some role in the lack of MGMT expression. Although we did not use the microdissection method, our results might be of less estimation because of the existence of nontumour DNA. If it is removed, further significant differences could be obtained.

### Methylation analysis of *hMLH1* promoter region in HCCs, noncancerous, and normal liver tissues

**Figure 5:**
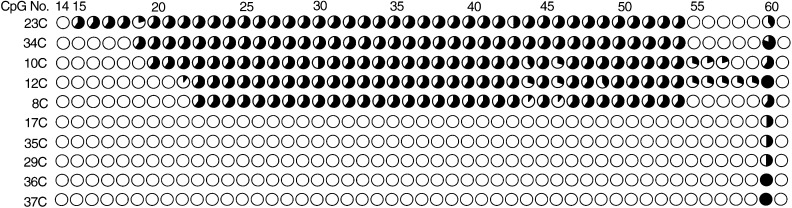
Detailed CpG (No. 14–61) methylation status of all analysed 67 CpGs within the *hMLH1* promoter region in 10 hMLH1-negative HCCs. Each circle graph represents the percentage of methylated clones (number of methylated clones/10 analysed clones×100).

The CpG island of *hMLH1* including 67 CpG sites is shown in Figure 2B. All of the CpG sites were also analysed by urea/bisulphite sequencing. In all, 10 hMLH1-negative HCCs were detected by immunohistochemistry, and five of these showed promoter hypermethylation (Figure 5

). The methylation patterns of these tumours were particular, that is, the middle portion of the CpG island was methylated and the methylation frequency of each CpG site in the region was approximately 60%. There was no methylated CpG in hMLH1-positive HCCs, noncancerous regions, and normal liver tissues (data not shown).

## DISCUSSION

To date, it is thought that inactivation of the *MGMT* gene is because of epigenetic changes such as DNA methylation (Esteller *et al*, 1999,^2001^) or some other unknown mechanisms because the genetic change is uncommon. Although the methylation of *MGMT* and *hMLH1* promoter was analysed previously in HCCs, the method used was mostly methylation-specific PCR (MSP) (Esteller *et al*, 2001). No study has identified critical sites by a survey of detailed methylation of the promoter regions of these genes. This is the first study to examine the detailed CpG methylation status of *MGMT* and *hMLH1* promoter regions in HCCs, their adjacent tissues, and normal liver tissues.

We compared MGMT-negative and -positive HCCs in order to determine the critical CpG site for the *MGMT* silencing. A subset of MGMT-negative tumours showed a high frequency of methylation within CpGs No. 1–9 and a wide extent of methylation beyond CpG No. 10. This evidence suggested that there were important CpG(s) for gene silencing in the promoter region. It was known that the *MGMT* enhancer, corresponding to our analysed CpGs No. 78–90, existed in the first intron. So far, the methylation of the enhancer (Harris *et al*, 1994) has not been reported in liver tumours by MSP (Esteller *et al*, 2001), and we also could not find any methylated CpG in the enhancer, except for sample 1C. It was reported that a single-site methylation upstream of the *p53* promoter, not in the enhancer (Lozano and Levine, 1991), reduced its expression during hepatocarcinogenesis *in vivo* (Pogribny *et al*, 2000). Therefore, it is possible that the methylation of specific CpG site(s) in the *MGMT* promoter, aside from the enhancer, could result in downregulation of *MGMT* gene expression in HCCs. Since we found that some MGMT-negative tumours did not show the very frequent and widespread methylation status, not only DNA methylation but also histone deacetylation, chromatin remodelling, post-transcriptional, and post-translational inactivation might be correlated with the MGMT deficiency. MGMT is converted to an inactive form after removing the methyl group from *O*^6^-methylguanine (Ishibashi *et al*, 1994). The inactivated MGMT is not degraded but remains in an immunoreactive state in normal cells (Liu *et al*, 2001), whereas it is degraded rapidly via the ubiquitin proteolytic pathway in tumour cells (Srivenugopal *et al*, 1996; Liu *et al*, 2001). In the light of this, some tumours that correspond to HCCs, 16C–28C, might be regarded as MGMT-negative because of the rapid degradation of the inactive form, although the gene might have been transcribed.

Our careful analysis revealed that both the frequency and extent of *MGMT* methylation increased along with pathological progression. Although little is known of the link between hepatitis viral infections and the methylation machinery of endogenous genes, our findings intimated that CpG methylation of the *MGMT* gene was associated with hepatitis viral infections. The levels of DNA methyltransferase mRNA in chronic hepatitis, cirrhosis, and HCCs associated with hepatitis viral infections increased compared with those in normal liver tissues (Sun *et al*, 1997). Human immunodeficiency virus induced the methylation of *interferon γ* (*IFNG*) through increased DNA methyltransferase activity (Mikovits *et al*, 1998). Therefore, CpG methylation of the *MGMT* promoter could possibly be caused by elevated DNA methyltransferase activity in hepatocarcinogenesis. In contrast to the *MGMT*, there was no methylation in *hMLH1*-positive HCCs, noncancerous, and normal liver tissues with or without hepatitis viral infection (data not shown), suggesting no relation between *hMLH1* promoter methylation and viral infections. The methylation mechanism for *hMLH1* should be different from that for *MGMT*. In the present study, the frequency of *hMLH1* promoter hypermethylation in HCCs was 10.9% (five out of 46) – half of hMLH1-negative HCCs – and consistent with the previous finding, 10.0% (two out of 20) (Esteller *et al*, 2001). There was no promoter methylation in the remaining five hMLH1-negative HCCs. In colon cancer, gene mutations were found in *hMLH1*-negative tumours without promoter hypermethylation (Cunningham *et al*, 1998). Although we did not investigate the gene mutations in those HCCs, genetic alterations would be involved in hMLH1-negative HCCs without promoter hypermethylation.
